# The Pros and Cons of the Prediction Game: The Never-ending Debate of Mortality in the Intensive Care Unit

**DOI:** 10.3390/ijerph16183394

**Published:** 2019-09-13

**Authors:** Piotr A. Fuchs, Iwona J. Czech, Łukasz J. Krzych

**Affiliations:** Department of Anaesthesiology and Intensive Care, School of Medicine in Katowice, Medical University of Silesia, 14 Medykow Street, 40752 Katowice, Poland

**Keywords:** intensive care unit, mortality, APACHE II, SAPS II, SOFA, prognosis

## Abstract

Background: The Simplified Acute Physiology Score (SAPS) II, Acute Physiology and Chronic Health Evaluation (APACHE) II, and Sequential Organ Failure Assessment (SOFA) scales are scoring systems used in intensive care units (ICUs) worldwide. We aimed to investigate their usefulness in predicting short- and long-term prognosis in the local ICU. Methods: This single-center observational study covered 905 patients admitted from 1 January 2015 to 31 December 2017 to a tertiary mixed ICU. SAPS II, APACHE II, and SOFA scores were calculated based on the worst values from the first 24 h post-admission. Patients were divided into surgical (SP) and nonsurgical (NSP) subjects. Unadjusted ICU and post-ICU discharge mortality rates were considered the outcomes. Results: Baseline SAPS II, APACHE II, and SOFA scores were 41.1 ± 20.34, 14.07 ± 8.73, and 6.33 ± 4.12 points, respectively. All scores were significantly lower among SP compared to NSP (*p* < 0.05). ICU mortality reached 35.4% and was significantly lower for SP (25.3%) than NSP (57.9%) (*p* < 0.001). The areas under the receiver-operating characteristic (ROC) curves were 0.826, 0.836, and 0.788 for SAPS II, APACHE II, and SOFA scales, respectively, for predicting ICU prognosis, and 0.708, 0.709, and 0.661 for SAPS II, APACHE II, and SOFA, respectively, for post-ICU prognosis. Conclusions: Although APACHE II and SAPS II are good predictors of ICU mortality, they failed to predict survival after discharge. Surgical patients had a better prognosis than medical ICU patients.

## 1. Introduction

There is an ongoing debate about mortality in Polish intensive care units (ICUs), a rate which seems to be high compared to Western European or North American countries [1]. This discrepancy has been extensively discussed in recent years [1,2,3,4,5].

The scoring systems assessing the severity of disease in intensive care units (ICU) have become a routine element of clinical assessment of short-term prognosis worldwide. The Simplified Acute Physiology Score (SAPS) II, Acute Physiology and Chronic Health Evaluation (APACHE) II, and Sequential Organ Failure Assessment (SOFA) are the most helpful tools for this purpose [6,7,8,9,10]. However, little is still known about their usefulness in predicting long-term outcomes, including post-ICU mortality [6,9,11]. Although several studies have investigated this issue, no clear conclusions have been formulated [12,13,14,15,16]. Unfortunately, one ought to be aware of the fact that the prognosis is based not only the severity of disease on admission by itself, but also a variety of demographic and clinical variables [12,13,14].

It is strongly recommended to verify the diagnostic accuracy of the scoring systems which may be specific for the population in which they are used. Therefore, they should be validated before their implementation in particular ICUs. In this study, we sought to evaluate the diagnostic accuracy of SAPS II, APACHE II, and SOFA in predicting ICU and post-ICU mortality of patients hospitalized in a tertiary university ICU.

## 2. Materials and Methods 

**Figure 1:**
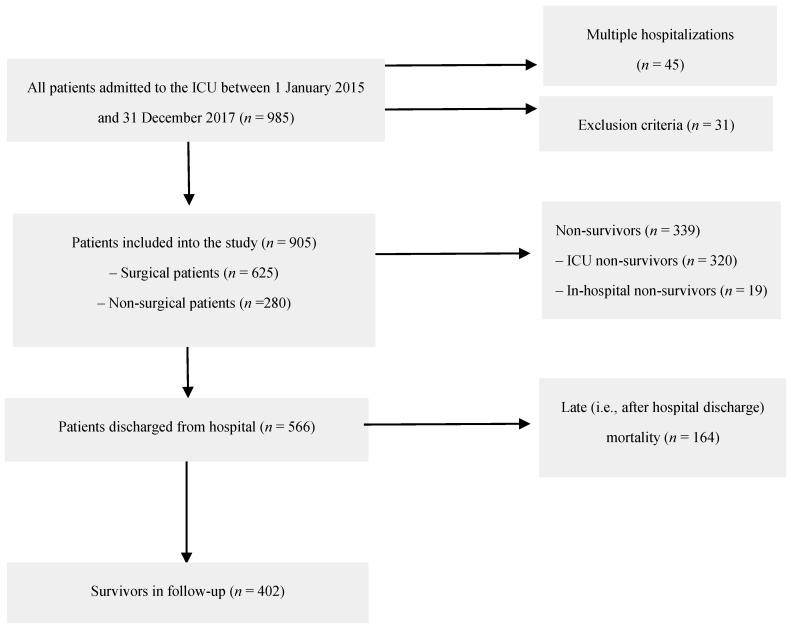
Patients’ flow chart.

All subsequent patients admitted to a mixed ICU between 1 January 2015 and 31 December 2017 (*n* = 936) were screened. Among them, 38 persons were hospitalized in the ICU more than once, which gave a total of 985 hospital stays. Excluded were patients at an age of <18 years old (*n* = 1), those with missing data in the hospital database (*n* = 10), those with no/incorrect national personal identity number or with unknown identity (*n* = 14), and those whose admission was only for organ procurement (*n* = 6). For patients who had been hospitalized more than once, their last admission was taken into account. Finally, 905 patients were included in the retrospective data analysis. Figure 1 presents the patients’ flow chart.

Demographic, clinical, and laboratory data were retrieved from medical records. SAPS II, APACHE II, and SOFA scores were calculated based on the worst values from the first 24 h post-admission [6,9,17]. The priority of ICU admission was assessed according to the recommendations of the Polish Society of Anesthesiology and Intensive Care, which are based on the recommendations of the Society of Critical Care Medicine (SCCM) [18]. Patients admitted according to the first priority were critically ill and required monitoring, therapy, and life support for organ failure that can be only provided in the ICU. Patients admitted according to the second priority were those who required intensive monitoring because they may have required invasive methods of treatment during hospitalization available only in the ICU. Patients admitted based on the third priority were those whose critical illness lowered their probability of recovery or survival (e.g., patients with metastatic cancer or terminal patients who required intensive pain management). The fourth priority included patients who should not have been hospitalized in the ICU (e.g., moribund patients or patients in good general condition). Patients were divided into surgical (SP) and nonsurgical (NSP) subjects. Unadjusted ICU and post-ICU discharge (i.e., among ICU survivors) mortality rates were calculated. Post-ICU discharge survival was evaluated based on data retrieved from the national PESEL (Polish Personal Identity Number) database. The cut-off point for follow-up observation was 10 August 2018.

The university Ethics Committee waived the requirement for informed consent due to the anonymous and non-interventional nature of the study (KNW/0022/KB/55/18).

Statistical analysis was performed using StatSoft Statistica version 13.0 software. Quantitative variables are presented as a mean and standard deviation (SD) or median and interquartile range (IQR). The qualitative variables are presented as an absolute value and/or percentage. Between-group differences for quantitative variables were verified using parametric (t-test or ANOVA) or non-parametric tests (U Mann-Whitney or Kruskal-Wallis), with previous verification of their distribution by the Shapiro-Wilk or Smirnov-Kolmogorov test. In the case of qualitative variables, the chi-square test or Fisher's exact test was used. A receiver-operating characteristic (ROC) curve analysis was used to assess the diagnostic accuracy of APACHE II, SAPS II, and SOFA scores. Kaplan-Mayer curves were drawn to assess long-term survival while a log-rank test was applied for curve comparisons. A *p* value of < 0.05 was considered significant.

## 3. Results

The median age of the patients was 62 (IQR 50–72) years while there were 493 (54.5%) females in the study group. There were 280 nonsurgical (30.9%) and 625 (69.1%) surgical patients, including 286 neurosurgical, 203 gastrointestinal, 94 gynecological, and 42 surgical subjects with other conditions. A total of 765 patients (84.5%) was admitted to the ICU based on the first SCCM priority, 88 (9.7%) based on the second priority, and 52 (5.8%) based on the third priority. None of the patients was admitted based on the fourth priority. 

Baseline SAPS II, APACHE II, and SOFA scores were 41.1 ± 20.34, 14.07 ± 8.73, and 6.33 ± 4.12 points, respectively. The scores differed significantly between SP and NSP, as well as between subjects classified based on the priority of admission (Table 1).

ICU mortality reached 35.4% (i.e., 320 deceased out of 905 ICU stays) and significantly differed between SP (*n* = 158, 25.3%) and NSP (*n* = 162, 57.9%) subgroups (*p* < 0.001). The scores were statistically significantly higher for non-survivors compared with ICU survivors, also in the SP and NSP subgroups (Table 2).

**Figure 2:**
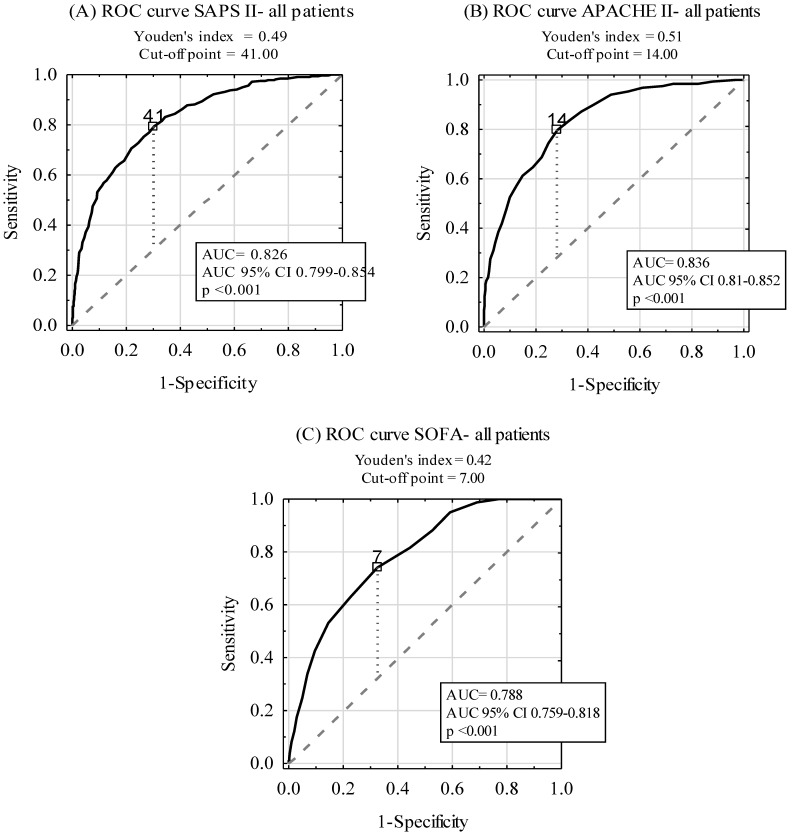
Diagnostic accuracy of SAPS II (**A**), APACHE II (**B**), and SOFA (**C**) scales in ICU mortality prediction.

ROC curves for ICU mortality prediction by SAPS II, APACHE II, and SOFA scales are depicted in Figure 2. The areas under the ROC curves (AUC) were 0.826, 0.836, and 0.788 for SAPS II, APACHE II, and SOFA scales, respectively. A subgroup analysis, including SP and NSP, is revealed in Table 3. Although there was no statistically significant difference between AUCs for SAPS II and APACHE II (*p* > 0.05), statistically significant differences between AUCs for SOFA and the two other scoring tools in the SP and NSP subgroups were found (*p* < 0.001).

The median observation of ICU survivors in the follow-up was 497 (IQR 247–848) days. Out of 585 ICU survivors, 183 (31.3%) persons died in the follow-up, giving an overall mortality rate of 53.5%. Out of 467 SP ICU survivors, 123 (22.7%) patients died, while out of 118 NSP ICU survivors, 60 (50.9%) subjects died post-ICU discharge (*p* < 0.001). 

**Figure 3:**
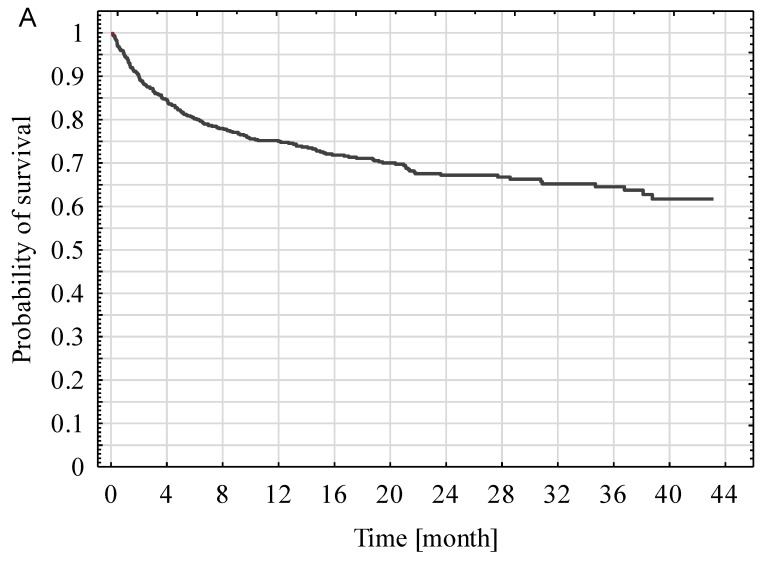

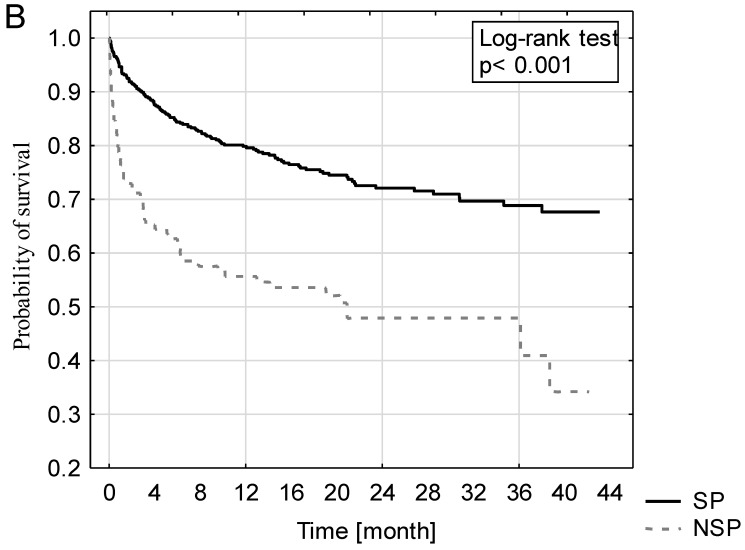
Kaplan-Mayer curves of all ICU survivors (**A**), including surgical and nonsurgical patients (**B**).

Kaplan-Mayer curves for post-ICU discharge survival for all patients, as well as in the SP and NSP subgroups, are presented in Figure 3. The survival rate of SP patients (67.6%) was significantly better compared with NSP patients (34.2%) (*p* < 0.001).

The investigated scores were statistically significantly higher in ICU survivors who died during the follow-up compared with those who were still alive (Table 4), also in the subgroup analysis of SP and NSP patients. 

**Figure 4:**
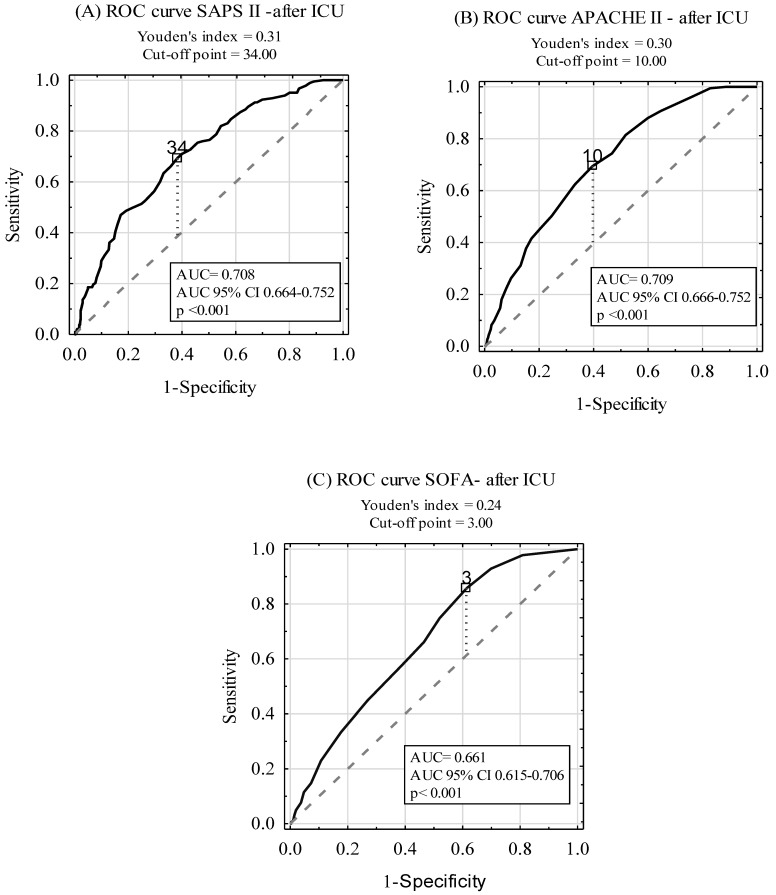
Diagnostic accuracy of SAPS II (**A**), APACHE II (**B**), and SOFA (**C**) scales in post-ICU mortality prediction in ICU survivors.

ROC curves for the prediction of post-ICU discharge mortality among ICU survivors by the SAPS II, APACHE II, and SOFA scales are shown in Figure 4. The AUCs were 0.708, 0.709, and 0.661 for SAPS II, APACHE II, and SOFA, respectively. A subgroup analysis, including SP and NSP patients, is presented in Table 5. Although there was no statistically significant difference between AUCs for SAPS II and APACHE II AUC (*p* > 0.05), statistically significant differences between AUCs for SOFA and the two other scoring tools (APACHE II, SAPS II) in the SP and NSP subgroups were found (*p* < 0.001).

## 4. Discussion

In this single-center observational study, we sought to investigate ICU and post-ICU mortality, with special attention given to the ability of SAPS II, APACHE II, and SOFA to predict the outcome. We found that the ICU mortality rate was 35.4%, which was lower than the value observed in the Silesia region (43.9%) [19], as well as in all Polish ICUs (42.0%) [1]. These values were much higher compared with those observed in other European countries, including 6.7% in Sweden, 8.5% in Germany, 8.9% in The Netherlands, 9.2% in Austria, 9.4% in Denmark, 10.7% in Norway, 11.2% in Spain, 14.9% in England, and 17.8% in Italy [1]. However, one ought to be aware of the fact that these marked differences are not due to insufficient care provided for the patients but rather the different profile of the patients admitted, different indications for admissions, and different organization of end-of-life care [3].

In our study, the baseline SAPS II, APACHE II, and SOFA scores and the predicted ICU mortality were as follows: 41.1 points (i.e., mortality rate of 33.6%), 14.07 points (mortality rate of 18.4%), and 6.33 points (mortality rate of 29.4%), respectively, which corresponded with the observed-to-predicted mortality rates of 1.05, 1.92, and 1.2, respectively. Of note is the fact that significantly higher scores were found for all three tools in non-survivors compared with survivors (*p* < 0.001), a phenomenon which had been previously confirmed by the other authors [6,20]. The cause of higher observed than expected mortality seems multifactorial. This may result from the difference in patients’ profiles in our mixed, heterogeneous unit. For instance, medical patients were confirmed to have higher mortality than surgical patients [21]. The number of patients admitted due to neurosurgical reasons reached 31.6% in our survey. In this unique group of subjects, the observed-to-predicted ratio was relatively high (i.e., SAPS II: 1.32, APACHE II: 3.36, SOFA: 1.63). The explanation of this discrepancy is quite simple: Although neurological subjects (i.e., those with brain trauma injury, subarachnoid hemorrhage, or post-operative brain edema) have often low scores in the first 24 h post-admission, their prognosis remains unfavorable. We also performed a deep sub-analysis of surgical subjects and found a confounding effect from the mode of the procedure, namely: All three models underestimated mortality in emergency patients, especially for APACHE II. SAPS II was more accurate for both elective and emergency surgery, with the observed-to-predicted ratios of 0.85 and 1.07, respectively. Furthermore, our analysis covered cardiac patients (8.18%) who were excluded from the original studies of APACHE, SAPS, and SOFA due to the noticeable difference between predicted and observed mortality [6,9,17] among them. Moreover, one ought to bear in mind that although 13% of variables were missing in the original APACHE II study, the subjects were included in the analysis. In our study, we excluded all subjects whose data were incomplete. 

Our observation of high accuracy (i.e., reaching 0.8 and more) of the scoring systems remains consistent with the previous studies performed worldwide [20,21,22,23]. Although the most powerful tool was APACHE II (AUC = 0.836), SAPS II was not superior in predicting ICU mortality (AUC = 0.826). Similar findings were reported by Giliani et al., who also confirmed that APACHE II was more reliable than SAPS II in surgical ICU patients [24]. In contrast, Sungurtekin et al. reported better prognostic accuracy for SAPS II than APACHE II for ICU subjects [25]. Aminiahidashti et al. demonstrated the comparable diagnostic accuracy of APACHE II and SAPS II in predicting 30-day ICU prognosis [16].

One ought to bear in mind that although ICU patients may recover from their critical illness, they are still at risk of subsequent mental and physical disabilities that cause deterioration of quality of life and higher post-discharge mortality. Therefore, accurate identification of patients who will stay at risk would enable medical services to implement proper screening and adequate treatment to reduce fatal complications after ICU hospitalization. Some of these deaths could have been prevented, while deterioration could probably have been avoided [13,26,27]. Campbell et al. and Yung-Che et al. emphasize that, in some cases, patients could have been discharged too early which resulted in short survival after ICU discharge or in ICU readmission [12,14]. Daly et al. reported that one-third of ICU patients in the UK are at increased risk of death after an ICU stay and that delaying their discharge by 48 hours could reduce the risk of death [28]. 

Although the primary goal of investigating the scoring systems was to estimate the risk of short-term mortality, we also aimed to verify whether these scales could be used to predict survival post-ICU discharge. We provided strong evidence that all three scoring systems failed to predict long-term mortality. These observations remain consistent with Campbell et al. who showed that routinely collected data cannot be used to produce clinically useful systems predicting death or readmission [13]. Other studies, which investigated long-term survival of ICU patients, also reported that there was no relation between the scores calculated on admission and death after ICU discharge [14]. The explanation is complex but understandable: The score is calculated based on the worst results during the first 24 h following admission and reflects the acute state of the illness only. The implemented post-admission treatment, intra-hospitalization complications, and post-discharge treatment influence the prognosis more than the basic APACHE, SAPS, or SOFA score. Therefore, we should rather focus on the scores calculated among ICU survivors on their discharge in order to try to predict the outcome. Lee et al. found that the discharge APACHE II score was a predictor of post-ICU mortality and readmission [12]. According to this observation, re-evaluation of the patient’s state at the end of the hospitalization would enable one to estimate the patient’s chances of survival in a more reliable way. What is more, other factors that are not included in the scoring systems, including patients’ behaviors and lifestyle, received medications, the quality of the follow-up care, and rehabilitation, influence patients’ chances to survive in a fundamental way. Therefore, more precise and reliable tools should be created in order to estimate the long-term prediction of the patients successfully discharged from the ICU. Further studies should be conducted concerning this subject.

Our study has some limitations. Firstly, the final results in the scoring systems may be affected by the confounding effect of the Glasgow Coma Score calculation. In many studies, patients receiving sedation on ICU admission were reported as GCS = 3 [16]. Secondly, our study was conducted as a single-center observation among a heterogeneous population, which may have center-specific diagnostic and therapeutic procedures. However, in order to overcome this drawback, we analyzed a large set of data for patients hospitalized in the Silesia region.

## 5. Conclusions

1. APACHE II and SAPS II are good predictors of the ICU mortality.
2. APACHE II, SAPS II, and SOFA fail to predict long-term mortality.
3. Surgical patients have better prognosis than medical ICU patients.
4. Further studies are needed to create reliable tools for the prognostication of critically ill patients successfully discharged from the ICU.

## Figures and Tables

**Table 1:** Simplified Acute Physiology Score (SAPS) II, Acute Physiology and Chronic Health Evaluation (APACHE) II, and Sequential Organ Failure Assessment (SOFA) scores.

Score		SAPS II	APACHE II	SOFA
Overall		41.1 ± 20.34	14.07 ± 8.73	6.33 ± 4.12
Reason for admission	Surgical	36.01 ± 18.86	11.67 ± 7.59	5.51 ± 3.9
	Nonsurgical	52.44 ± 18.9	19.44 ± 8.72	8.15 ± 4.03
	‘*p*’	<0.001	<0.001	<0.001
Priority of admission	First	42.47 ± 18.73	14.45 ± 8.26	6.7 ± 3.83
	Second	15.89 ± 9.85	4.88 ± 3.47	0.92 ± 1.84
	Third	63.54 ± 16.44	24.0 2± 7.62	9.92 ± 3.04
	‘*p*’	<0.001	<0.001	<0.001

**Table 2:** SAPS II, APACHE II, and SOFA scores and intensive care unit (ICU) mortality.

Score		ICU Survivors	ICU Non-survivors	‘*p*’
SAPS II	Overall	32.98 ± 16.38	56.04 ± 18.29	<0.001
	Surgical	30.37 ± 15.33	52.68 ± 18.48	<0.001
	Nonsurgical	43.01 ± 16.53	59.31 ± 17.54	<0.001
APACHE II	Overall	10.44 ± 6.5	20.71 ± 8.37	<0.001
	Surgical	9.3 ± 5.77	18.65 ± 8.01	<0.001
	Nonsurgical	14.96 ± 7.25	22.71 ± 8.24	<0.001
SOFA	Overall	4.86 ± 3.6	9 ± 3.62	<0.001
	Surgical	4.51 ± 3.49	8.44 ± 3.53	<0.001
	Nonsurgical	6.24 ± 3.76	9.55 ± 3.63	<0.001

**Table 3:** Diagnostic accuracy of SAPS II, APACHE II, and SOFA scales in ICU mortality prediction among surgical and nonsurgical patients.

Score	Surgical Patients		Non-surgical Patients	
	AUC (95%CI)	‘*p*’	AUC (95%CI)	‘*p*’
SAPS II	0.826 (0.788–0.863)	<0.001	0.742 (0.686–0.799)	<0.001
APACHE II	0.836 (0.801– 0.872)	<0.001	0.748 (0.691–0.804)	<0.001
SOFA	0.781 (0.743–0.82)	<0.001	0.739 (0.679–0.798)	<0.001

**Table 4:** SAPS II, APACHE II, SOFA scores and post-ICU mortality in ICU survivors.

Score		ICU Survivors Remaining Alive During Follow-up	ICU Survivors Who Died During Follow-up	‘*p*’
SAPS II	Overall	29.29 ± 15.6	40.9 ± 15,21	<0.001
	Surgical	28.11 ± 14.83	36.71 ± 15.03	<0.001
	Non-surgical	36.31 ± 18.19	49.48 ± 11.64	<0.001
APACHE II	Overall	9.03 ± 6.1	13.55 ± 6.29	<0.001
	Surgical	8.51 ± 5.66	11.52 ± 5.51	<0.001
	Non-surgical	12.12 ± 7.58	17.7 ± 5.77	<0.001
SOFA	Overall	4.25 ± 3.55	6.2 ± 3.39	<0.001
	Surgical	4.09 ± 3.47	5.7 ± 3.3	<0.001
	Non-surgical	5.22 ± 3.9	7.22 ± 3.39	0.002

**Table 5:** Diagnostic accuracy of SAPS II, APACHE II, and SOFA scales in post-ICU mortality prediction among surgical and nonsurgical ICU surviving patients.

Score	Surgical Patients		Non-surgical Patients	
	AUC (95%CI)	‘*p*’	AUC (95%CI)	‘*p*’
SAPS II	0.659 (0.605–0.713)	<0.001	0.719 (0.624–0.814)	<0.001
APACHE II	0.666 (0.614–0.717)	<0.001	0.723 (0.63–0.817)	<0.001
SOFA	0.641 (0.587–0.694)	<0.001	0.663 (0.564–0.762)	0.001

## Abbreviations

ICU	Intensive Care Unit
SAPS	Simplified Acute Physiology Score
APACHE	Acute Physiology and Chronic Health Evaluation
SOFA	Sequential Organ Failure Assessment
SCCM	Society of Critical Care Medicine
SP	surgical patients (subjects)
NSP	non-surgical patients (subjects)
